# A Mechanistic Approach Toward Enhanced Remediation Potential of Thiacloprid by Zero-Valent Iron/Biochar Supplemented with Organic Acids

**DOI:** 10.3390/nano15080570

**Published:** 2025-04-08

**Authors:** Lin Pan, Shuai Qu, Longfei Liu

**Affiliations:** 1 Criminal Investigation College, People’s Public Security University of China, Beijing 100038, China; ppsuclin@163.com; 2 College of Environmental Science and Engineering, Yangzhou University, Yangzhou 225127, China; mz120241367@stu.yzu.edu.cn; 3 Key Laboratory of Arable Land Quality Monitoring and Evaluation, Ministry of Agriculture and Rural Affairs, Yangzhou University, Yangzhou 225127, China

**Keywords:** zero-valent iron, thiacloprid, humic acid (HA), oxalic acid (OA), electron transfer

## Abstract

The excessive levels of neonicotinoid insecticides, particularly thiacloprid (THI), in the environment have become a significant threat to ecosystems. This study investigates the catalytic degradation of THI using pinewood biochar (PBC), zero-valent iron (ZVI), and ZVI/PBC composite, with a particular focus on the reaction activity modulation mediated by organic acids (humic acid: HA and oxalic acid: OA). Reductive dechlorination dominated THI degradation as observed by Cl^−^ release kinetics. Compared to HA (39.73%), the OA (73.44%) addition markedly increased the THI removal efficiency by ZVI/PBC, which alone has a lower removal efficacy, i.e., 37.29%. The increase in the THI removal rate was attributed to its enhanced electron transfer capacity. As confirmed by electrochemical characterization, the addition of organic acids promotes electron transfer between THI and catalysts (ZVI, PBC, or ZVI/PBC), thereby improving the removal efficiency of THI. XRD/XPS analyses elucidated that OA preferentially converted passivating Fe_2_O_3_/Fe_3_O_4_ on ZVI/PBC to reactive FeOOH and formed electron-conductive Fe–COO bonds, thereby suppressing oxide layer formation. PBC amplified these effects through ZVI dispersion and electron shuttling, reducing aggregation-induced activity loss. These findings provide a mechanistic framework for optimizing ligand-engineered iron composites, offering practical strategies to enhance pesticide remediation efficiency in organic acid-rich environmental systems.

## 1. Introduction

Neonicotinic insecticides are a kind of neurotoxic insecticide widely used globally [1] based on higher selectivity and effectiveness against certain destructive crop pests. These pesticides, particularly thiacloprid, can compete with the natural neurotransmitter acetylcholine and disrupt signal transmission, impacting insects’ central nervous system [2]. The pronounced lethality of neonicotinoids toward target pests, coupled with their relatively low mammalian toxicity, has led to their overapplication in the agricultural soil–plant system, resulting in substantial residues within soil, aquatic ecosystems, and agricultural produce [3]. It is estimated that during application, about 5% of the active ingredients are absorbed by crops, whereas the vast majority (~90%) reside in the soil from where they might be transported into aquatic systems such as lakes, rivers, or groundwater via runoff from precipitation [4,5]. The widespread contamination of thiacloprid poses significant risks to human health through thiacloprid-laced dietary exposure, thus contaminating the food chain [6]. Therefore, the environmental contamination attributable to neonicotinoid pesticides has emerged as a critical global concern. The application of THI (chloronicotinic insecticide) in agricultural settings effectively controls chewing and sucking pests due to its strong contact, stomach toxicity, and nervous systemic disruption activity [7]. Owing to THI’s high solubility and hydrophilicity, which facilitates its leaching into rivers and groundwater through irrigation and rainfall, urgent measures for THI remediation from water sources are necessitated [8].

Zero-valent iron (ZVI) has emerged as a sustainable sorbent for both organic and inorganic pollutant remediation, leveraging its reductive capacity and adsorption affinity [9,10]. Previous studies have shown that THI removal by ZVI proceeds via reductive dechlorination, where surface-bound Fe^0^ donates electrons to THI through sequential complexation, electron transfer, and product dissociation [11]. However, ZVI’s practical application is restricted either via particle aggregation (driven by van der Waals forces) or rapid surface passivation through metastable iron oxide formation [12,13]. The surface modifications of ZVI with sulfidation and surfactant coating might resolve ZVI limitation, its associated environmental risks (e.g., sulfide leaching), and activity obstacles (e.g., active site occlusion) [14,15].

Biochar-supported ZVI composites (ZVI/BC) offer a promising alternative, synergizing ZVI’s reactivity with biochar’s structural and functional advantages that might enhance ZVI dispersion, minimize oxidation via oxygen barrier effects, and facilitate electron transfer through redox-active functional groups (e.g., phenolic donors and quinonic acceptors) [16,17]. Critically, biochar’s oxygen-containing groups chelate dissolved Fe^2^⁺/Fe^3^⁺, inhibiting oxide passivation and sustaining ZVI reactivity with biochar enrichment [18]. As reported by one study, the application of ZVI (1 mg/g) removed 89% and 86% of imidacloprid and thiamethoxam, respectively, and both are neonicotinoid pesticides commonly used in agriculture [19]. Moreover, biochar promotes several degradation pathways, such as Fenton reactions, photocatalysis, persulfate oxidations, and biodegradation, predominantly through non-radical (like O_2_ and electron transfer) and radical (like SO_4_·^−^, ·OH, and ·O_2_^−^) processes [20,21].

Biochar aids in organic matter complexation as organic matter provides more sorption sites, thereby affecting the adsorption and reduction in pollutants by ZVI. On the other hand, organic matter has many surface functional groups like carboxylic, phenols, thiols, hydroxyls, etc., which can modulate the THI removal mechanisms [20,22,23,24]. To disentangle these effects, this study investigates humic acid (HA) and oxalic acid (OA)—model compounds representing high- and low-molecular-weight OM—in ZVI and ZVI/BC systems. We hypothesize that OA’s smaller molecular size enhances Fe-carboxylate coordination and electron shuttling, while HA’s steric hindrance limits its catalytic potential. Electrochemical and surface morphology analyses were used to verify the above hypothesis. Therefore, this study advances predictive frameworks for ligand-engineered Fe systems, addressing critical gaps in sustainable pesticide remediation technologies.

## 2. Materials and Methods

### 2.1. Reagents

Pinewood was purchased from the Huifeng Straw Processing Plant in Lianyungang (Jiangsu, China). Thiacloprid (THI) was supplied by the Wuhan Yuancheng Technology Development Company (Wuhan, China). Ferric chloride hexahydrate (FeCl_3_·6H_2_O), oxalic acid (OA), sodium borohydride (NaBH_4_), sodium sulfate (Na_2_SO_4_), acetonitrile (CH_3_CN), and formic acid (CH_2_O_2_) were obtained from Sinopharm Chemical Reagent Co., Ltd. (Shanghai, China). Humic acid (HA) was purchased from Aladdin Bio-Chem Technology Co. (Shanghai, China). Deionized (DI) water (18.25 MΩ cm^−1^) was used for the preparation of aqueous solutions.

### 2.2. Preparation of Materials

#### 2.2.1. Pinewood Biochar (PBC) Synthesis

Pinewood shreds were placed in a porcelain boat and subjected to pyrolysis in a tubular furnace under anoxic conditions (with continuous nitrogen purging), and the heating rate was set at 5 °C/min, reaching a final temperature of 500 °C, which was maintained for 2 h. The resulting material (pinewood biochar; hereafter referred to as PBC) was washed three times with a mixture of deionized water and ethanol (1:1 *v*/*v*), followed by drying in an oven at 60 °C for 24 h. It was then ground into a fine powder and stored for further use.

#### 2.2.2. ZVI/PBC Composite Fabrication

To prepare the ZVI/PBC composite, 1 g of PBC and 0.968 g of FeCl_3_·6H_2_O were separately dispersed in 100 mL of deionized water and sonicated for 30 min. In parallel, 0.529 g of NaBH_4_ was dissolved in 120 mL of deionized water. With argon gas supplementation, both solutions were mixed dropwise, and after the addition, the mixture was further sonicated for 30 min to ensure a complete reaction. The resulting product, designated as ZVI/PBC, was washed three times with ethanol, dried in a vacuum oven at 60 °C for 24 h, and collected for subsequent analysis.

#### 2.2.3. ZVI Preparation

The preparation procedure for zero-valent iron (ZVI) was similar to that of ZVI/PBC, with the exception that PBC was not included in the synthesis process.

### 2.3. Characterization

Elemental composition, morphology, and the associated surface functional groups of three materials were characterized using X-ray photoelectron spectroscopy (XPS, ESCALAB 250Xi, Thermo Fisher Scientific, Waltham, MA, USA). The crystal structures of the synthesized materials were acquired using an X-ray diffraction (XRD) analysis with a Cu Kα radiation source (λ = 1.5418 Å) in the diffraction angles between 10 and 80° (Ultima IV X-Ray Diffractometer, Rigaku Corporation, Tokyo, Japan). The electrochemical properties of the materials were explored with an electrochemical workstation (CHI660e, CH Instruments Inc., Austin, TX, USA). The morphology and structure of materials through field emission scanning electron microscopy (S-4800 II, Hitachi, Tokyo, Japan) were analyzed. ZVI/PBC was characterized by high-resolution transmission electron microscopy (HR-TEM) (Tecnai G2 F30 S-TWIN, FEI, Hillsboro, OR, USA). The determination of Cl^−^ concentration in solution was obtained by ion chromatography (ICS-2000, Thermo Fisher Scientific, Waltham, MA, USA).

### 2.4. Removal Experiment of THI

A THI solution with an initial concentration of 20 mg/L was prepared, and 12.5 mg of pristine PBC material, 2.5 mg of zero-valent iron (ZVI), and 15 mg of ZVI/PBC composite were separately added into a 50 mL reactor, respectively. The mass ratios of ZVI to PBC in the ZVI/PBC composite were maintained equivalent to those of the individual controls. Further, treatments with 1.2 mg of HA (40 mg/L) and 1.2 mg of OA were also prepared.

To each centrifuge tube containing the materials (PBC, ZVI, ZVI/PBC, HA, and OA), 30 mL of a 20 mg/L THI solution was added and placed in a constant temperature water bath shaker (25 ± 1 °C) for the course of incubation. Samples were collected at predetermined time intervals (30, 60, 120, 240, 480, 720, and 1440 min) and filtered through 0.22 μm filters to remove any particulate matter.

THI concentrations were quantified via high-performance liquid chromatography (HPLC, Agilent 1260, Santa Clara, CA, USA), equipped with a C18 reverse-phase column (4.6 × 250 mm, 5 μm) and UV detector (λ = 254 nm). The mobile phase comprised a 40:60 (*v*/*v*) mixture of 0.1% formic acid in water and acetonitrile at a flow rate of 1.0 mL/min, with a 20 μL injection volume. Calibration curves (R^2^ > 0.999) were constructed using THI standards.

All experiments were conducted in triplicate under dark conditions to prevent photolytic interference. Blank controls (THI solution without materials) and matrix controls (materials without THI) were included to account for background adsorption and abiotic degradation.

## 3. Results and Discussion

### 3.1. Characterization

**Figure 1:**
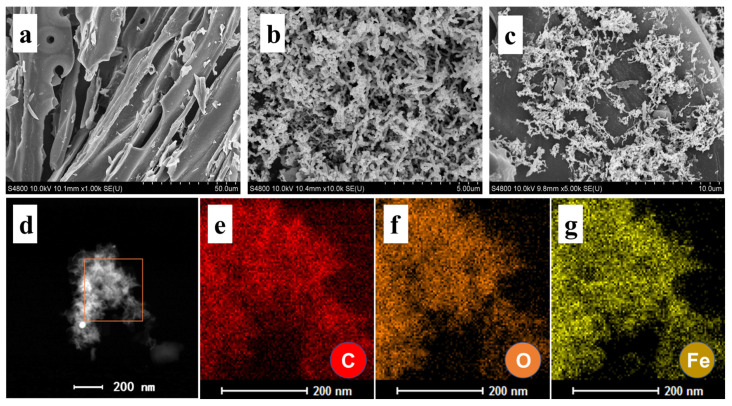
Scanning electron microcopy (SEM) (**a**–**c**) images of PBC, ZVI, and ZVI/PBC; and (**d**–**g**) EDS elemental mapping images of ZVI/PBC.

The morphological analysis reveals distinct structural characteristics of the synthesized materials (Figure 1). PBC exhibits a layered architecture with irregular microporous features (Figure 1a), while ZVI displays a chain-like morphology with pronounced particle aggregation (Figure 1b) [25]. In contrast, the ZVI/PBC composite demonstrates the effective dispersion of ZVI nanoparticles within the porous matrix and surface of PBC (Figure 1c), confirming successful heterostructure fabrication [26]. Notably, the aggregation observed in standalone ZVI is markedly mitigated in the composite, underscoring the role of PBC as a stabilizing substrate for ZVI dispersion. Energy-dispersive X-ray spectroscopy (EDS) elemental mapping further corroborates the homogeneous distribution of iron across the PBC surface, validating the uniformity of ZVI integration (Figure 1d–g).

**Figure 2:**
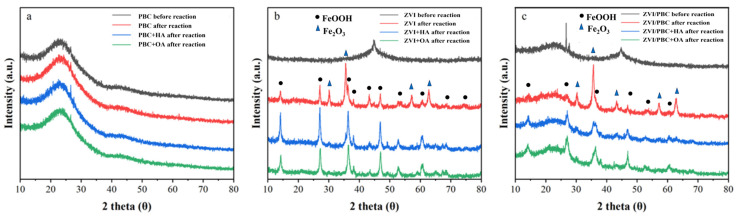
XRD patterns of (**a**) PBC, (**b**) ZVI, and (**c**) ZVI/PBC with the addition of organic acids before and after reaction.

The crystalline phases of the synthesized materials were explored using the XRD pattern (Figure 2). The XRD pattern of PBC (Figure 2a) features a broad amorphous carbon peak centered at 2θ = 23.5°, consistent with its graphitic carbon-dominated structure. Both pure ZVI and ZVI/PBC (Figure 2b,c) exhibit a characteristic diffraction peak at 2θ = 44.8°, corresponding to the (110) lattice plane of zero-valent iron (PDF#06-0696). The preservation of this peak in the composite, without detectable phase shifts or attenuation, confirms the structural retention of ZVI after immobilization on PBC [27].

### 3.2. THI Remediation Potential

**Figure 3:**
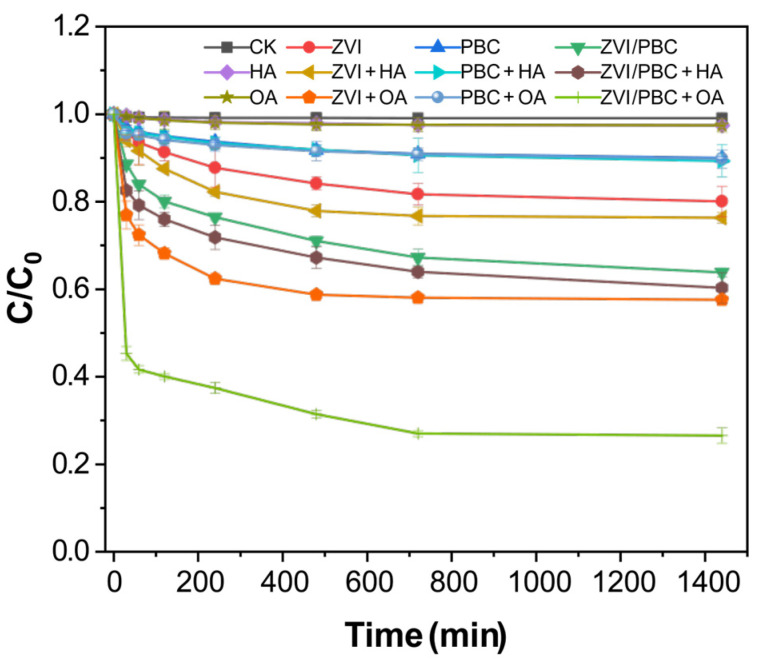
The effect of organic matter on the removal of THI by PBC, ZVI, and ZVI/PBC ([HA] = [OA] = 0.04 g/L, [ZVI] = [PBC] = [ZVI/PBC] = 0.2 g/L, equilibrium pH = 6.2, and T = 25 ± 1 °C).

The thiacloprid removal capacities of PBC, ZVI, and ZVI/PBC were comparatively evaluated based on aqueous-phase THI depletion kinetics (Figure 3). After 24 h, the removal efficiencies of THI reached 9.98% (PBC), 19.93% (ZVI), and 36.15% (ZVI/PBC), respectively. Notably, the theoretical additive efficiency of PBC and ZVI (29.91%) was significantly lower than the observed ZVI/PBC performance (36.15%), demonstrating a synergistic interaction between ZVI and PBC and interlinked with the passivation of ZVI and the electron transfer between ZVI and PBC. Also, rich oxygen-containing functional groups on the surface of PBC complexed with the Fe ions generated by ZVI passivation, forming a protective corrosion layer (rust) that inhibited ZVI reoxidation [16]. Moreover, the PBC-mediated dispersion of ZVI nanoparticles reduced aggregation and enhanced electron transfer efficiency, which accelerated the dichlorination kinetics of THI [28] as PBC acts as an electron shuttle, promoting electron transport between ZVI and THI.

Control experiments with HA and OA demonstrated non-significant least THI removal, i.e., 2.56% and 2.57%, respectively, compared to the blank. With the addition of HA and OA to the PBC treatment, a slight increase in the THI removal efficiency was observed by 10.68% and 10.24%, respectively, compared to PCB. This indicated that HA and OA have the least removal efficiency of THI, while, with the addition of HA and OA, a slight increase was observed in the removal of THI by PBC. Adsorption was the primary mode of THI removal by PBC [22,29,30]. In contrast, the ZVI system showed varying degrees of improvement in THI removal upon HA addition by 23.65%, and it was 42.41% for ZVI+OA, which represented 3.72% and 22.48% increments over ZVI.

The synergistic effect was amplified in ZVI/PBC composites, with THI removal dramatically rising to 39.73% (ZVI/PBC+HA) and 73.44% (ZVI/PBC+OA), compared to ZVI/PBC. This differential enhancement highlights the superior catalysis of OA compared to HA. The pronounced OA-driven performance may stem from dual mechanisms, including (i) that the electron transfer kinetics at the ZVI/PBC-THI interface were enhanced by the addition of OA, and (ii) the addition of OA inhibited the iron oxide passivation layer through chelation and maintained the reactivity of ZVI [23]. The primary reason that HA is less effective than OA in enhancing the removal rate of THI is due to its high molecular weight and complex structure [31]. These characteristics can encapsulate PBC or ZVI, thereby hindering the effective binding of THI molecules to them. Consequently, the addition of HA has a limited effect on the removal efficiency of THI [32].

The THI removal efficiency of the ZVI/PBC-OA system outperforms most reported values for neonicotinoid degradation using iron-based composites (Table 1). This indicates that the material can efficiently degrade THI without additional conditions such as light, ultraviolet (UV), or periodate and has significant environmental friendliness. OA significantly enhances the activity of ZVI by enhancing electron transfer and inhibiting iron oxide passivation. In contrast, other studies often require additional oxidants or complex conditions. This research method is simple and efficient, providing a low-cost and easy-to-operate solution for practical environmental remediation.

### 3.3. Solution Phase Dissolved Cl^−^ Concentration Dynamics

**Figure 4:**
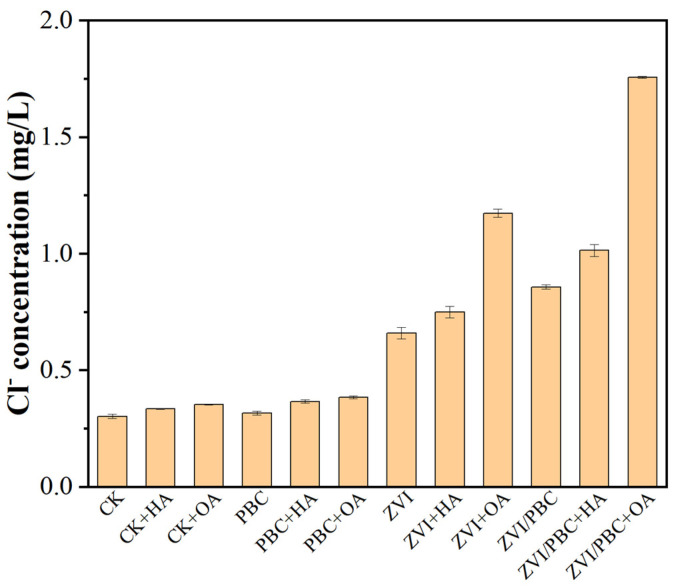
Concentration of Cl^−^ in solution after PBC, ZVI, and ZVI/PBC reactions.

The dechlorination capacity of ZVI-based systems was quantitatively assessed through chloride ion (Cl^−^) release analysis, serving as a critical indicator of THI degradation pathways. Given that the initial THI concentration was 20 mg/L (containing ~2.81 mg/L of theoretically releasable Cl^−^), systematic comparisons were conducted across treatment conditions (Figure 4). In the blank and control, background Cl^−^ concentration remained at 0.30 mg/L, with minimal increases observed in HA (0.33 mg/L) and OA (0.35 mg/L) additions, confirming their negligible dechlorination activity owing to structural complexities. Similarly, PBC-dominated treatments exhibited marginal Cl^−^ accumulation by 0.32–0.38 mg/L in PBC, PBC+HA, and PBC+OA, respectively, consistent with adsorption-mediated THI removal mechanisms [29]. ZVI systems demonstrated enhanced dechlorination activity, releasing 0.66 mg/L of Cl^−^ (23.5% of the theoretical maximum). This efficiency was enhanced by HA (0.75 mg/L, 26.7%) and significantly increased by OA (1.17 mg/L, 41.6%), attaining a 2-fold dechlorination capacity compared to pristine ZVI. The ZVI/PBC composite increased the dechlorination potential by 30.6% (0.86 mg/L Cl^−^), which further increased to 1.01 mg/L (35.9%) and 1.76 mg/L (62.6%) with HA and OA additions, respectively. This indicated that ZVI had a good reducing dechlorination effect on THI, and the addition of organic matter, especially OA, can improve the dechlorination efficiency of ZVI. Also, ZVI/PBC has better performance in dechlorination, indicating that reducing dechlorination is the main way for ZVI/PBC to remove THI [30].

### 3.4. Mechanisms of THI Degradation

The degradation of THI first involves adsorption, where THI molecules are adsorbed on the surface of ZVI, PBC, or ZVI/PBC. This is because these materials have specific surface properties and functional groups that can interact with THI. Subsequently, under the action of zero-valent iron in ZVI, THI undergoes a reduction reaction, mainly through electron transfer processes. In this process, ZVI acts as an electron donor, transferring electrons to THI molecules adsorbed on its surface, thereby triggering changes in its chemical structure, and achieving degradation.

**Figure 5:**
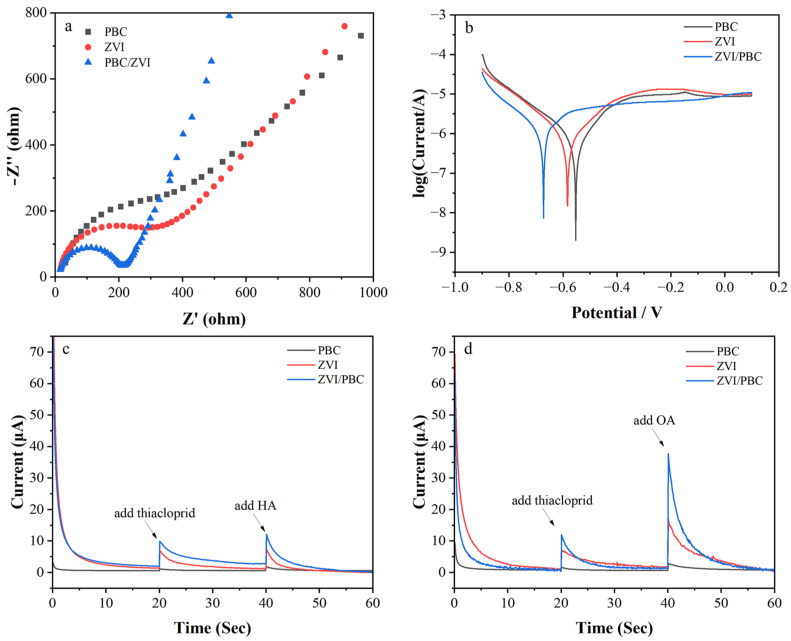
Electrochemical analysis of PBC, ZVI, and ZVI/PBC: (**a**) electrochemical impedance spectroscopy; (**b**) Tafel corrosive curves; (**c**) *i*–*t* response curves of added HA; (**d**) *i*–*t* response curves of added OA.

Electrochemical characterization was employed to elucidate the role of electron transfer in THI removal by ZVI/PBC composites. Nyquist plots (Figure 5a) revealed a reduced semicircle radius for ZVI/PBC compared to ZVI and PBC, indicating enhanced charge transfer efficiency due to PBC-mediated ZVI dispersion and interfacial conductivity optimization [37], attributed to PBC’s graphitic carbon framework and its ability to stabilize dissolved Fe^2+^/Fe^3+^ ions via chelation, thereby mitigating oxide layer formation. Tafel polarization curves (Figure 5b) further demonstrated a cathodic shift in corrosion potential for ZVI/PBC (−0.67 V) relative to ZVI (−0.58 V) and PBC (−0.55 V), confirming its superior electron-donating capacity and thermodynamic propensity for reductive reactions as reported by Mortazavian, An, Chun, and Moon [38]. Chronoamperometric profiling (Figure 5c–d) quantified interfacial electron flux under operational conditions. Upon immersion in the THI solution, ZVI/PBC exhibited a 404% current increase (from 1.96 μA to 9.87 μA), significantly exceeding PBC (97%: from 0.58 to 1.14 μA) and ZVI (398%: from 1.38 to 6.87 μA). This disparity highlights PBC’s dual role as both a conductive substrate and electron shuttle, synergistically amplifying ZVI’s redox activity [39].

Therefore, the addition of HA and OA enhances the electron transfer capacity of ZVI and ZVI/PBC. The large number of surface functional groups present on the surface of HA and OA facilitates electron shuttling among THI and ZVI or ZVI/PBC. Meanwhile, HA and OA can combine with iron ions to inhibit the deposition of iron oxides on the surface of ZVI and ZVI/PBC, thereby promoting efficient electron transfer [40]. In addition, HA and OA stimulate the oxidation of ZVI and ZVI/PBC, leading to an increased production of Fe(^2+^) and consequently enhancing electron transport efficiency. The compact structure of OA and its dual carboxyl functional groups can form tight Fe–COO bonds with ZVI. This enhances the electron transfer between THI and the material. This is manifested as a significant increase in current (Figure 5d). The comparatively lower enhancement effect of HA relative to OA may be attributed to its higher molecular weight, facilitating its adsorption onto the ZVI surface and subsequently impeding electron transfer processes [41].

To explore the impact of iron oxide passivation on electron transfer dynamics, post-reaction mineralogical phase analysis was conducted via XRD (Figure 2b). ZVI exhibited extensive oxidation post-reaction and exhibited characteristic peaks of γ-FeOOH (PDF#44-1415) and α-Fe₂O (PDF#33-0664). This dual-phase oxidation confirms ZVI passivation during THI degradation, consistent with its diminished electron transfer capacity. However, HA- or OA-amended ZVI systems exclusively retained γ-FeOOH signatures, with a complete suppression of α-Fe₂O₃ formation. This selective phase regulation aligns with organic acids’ chelation capacity, which stabilizes metastable Fe(^2+^/^3+^)-oxyhydroxides over crystalline Fe_2_O_3_, thereby mitigating passivation and preserving ZVI reactivity [42].

In ZVI/PBC composites, post-reaction XRD revealed metastable magnetite (Fe_3_O_4_, PDF#19-0629) alongside γ-FeOOH. The coexistence of Fe_3_O_4_ (a mixed-valence Fe(^2+^)/Fe(^3+^) phase) and γ-FeOOH suggests PBC-mediated redox buffering, where carbon matrices facilitate electron redistribution to stabilize intermediate oxidation states [43,44]. The HA/OA addition further modulated phase composition, suppressing Fe₃O₄ crystallization and promoting amorphous γ-FeOOH dominance, a critical factor in maintaining sustained electron transfer efficiency, which is due to the semiconducting properties of FeOOH imposing a lower electron transfer barrier compared to fully oxidized Fe_2_O_3_/Fe_3_O_4_ [42].

**Figure 6:**
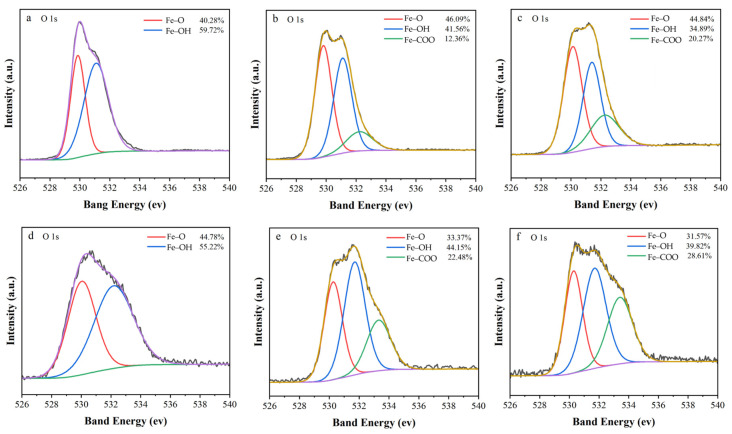
High-resolution XPS spectra of O 1s (**a**) after ZVI reaction; (**b**) after adding HA reaction in ZVI; (**c**) after adding OA reaction in ZVI; (**d**) after ZVI/PBC reaction; (**e**) after adding HA reaction in ZVI/PBC; (**f**) after adding OA reaction in ZVI/PBC.

XPS was employed to unveil organic acid-mediated surface transformations impacting electron transfer dynamics (Figure 6). The O 1s spectrum of post-reaction ZVI (Figure 6a) exhibited two dominant peaks at 529.86 eV (Fe–O, 40.28%) and 531.09 eV (Fe–OH, 59.72%), characteristic of a mixed Fe^2+^/Fe^3+^ oxide–hydroxide passivation layer [45]. HA and OA addition induced a third peak at 532.23 eV (Figure 6b–c), assigned to carboxylate-bound iron (Fe–COO). The carboxyl functional groups contained in HA and OA can be adsorbed and combined by ZVI to form typical Fe–COO. Specifically, the addition of HA introduced an Fe–COO peak at 532.23 eV (12.36%), accompanied by an Fe–O increase (46.09%) and an Fe–OH decrease (41.56%), whereas it amplified Fe–COO contribution to 20.27% after the addition of OA, with Fe–O at 44.84% and Fe–OH reduced to 34.89%. These changes indicate that organic acids displace surface hydroxyl groups through monodentate coordination [22], forming iron–carboxylate complexes that inhibit oxide polycondensation, preserve electron-conductive Fe^0^/Fe^2+^ domains [46], and enhance interfacial charge transfer via delocalized carboxylate π-systems [47].

In ZVI/PBC composites, the addition of HA and OA dramatically increased Fe–COO formation to 22.48% and 28.61%, respectively, while reducing Fe–O (33.37% HA and 31.57% OA) and Fe–OH (44.15% HA and 39.82% OA). The 2.1–3.5-fold higher Fe–COO content compared to ZVI underscores PBC’s dual role in enhancing ZVI dispersion to facilitate iron–organic coordination and act as an electron shuttle to accelerate Fe^0^ oxidation, thereby increasing soluble Fe^2+^ availability for complexation [48]. Notably, OA’s superior performance in Fe–COO generation (28.61% vs. HA’s 22.48%) correlates with its smaller molecular size, enabling deeper surface penetration and stronger chelation. The labile nature of Fe–COO bonds, compared to stable Fe–OH/Fe–O species, reduces electron transfer barriers, consistent with the enhanced chronoamperometric currents observed in OA-modified systems. These findings highlight the critical role of organic acid-induced surface reconstruction in optimizing electron transfer efficiency and mitigating passivation in ZVI-based remediation systems [43,49,50].

### 3.5. Future Research and Challenges

The scalability and practical application of the ZVI/PBC-organic acid system face several challenges that warrant consideration. Material stability remains a critical concern: while PBC mitigates ZVI oxidation by chelating Fe^2+^/Fe^3+^ and suppressing oxide formation (XRD/XPS data), long-term reactivity under aerobic or fluctuating pH conditions needs further validation. For instance, repeated redox cycles in natural environments may accelerate Fe^0^ consumption or biochar degradation, necessitating periodic material replenishment. Cost-effectiveness is another pivotal factor. Although pinewood biochar and ZVI are low-cost precursors (~50–100/ton for biochar; 50–100/ton for biochar; 1–2/kg for ZVI), the energy-intensive pyrolysis (500 °C) and NaBH_4_-driven ZVI synthesis could escalate operational expenses. A comparative analysis against conventional methods (e.g., activated carbon adsorption or Fenton oxidation) is essential to justify economic viability.

In complex environmental matrices, competing ions (e.g., Cl^−^ and SO_4_^2−^), dissolved organic matter, and variable pH may hinder THI degradation. For example, high salinity could promote ZVI corrosion via chloride-induced pitting, while humic substances might compete with OA for Fe-binding sites, reducing catalytic efficiency. Pilot-scale testing in real wastewater or soil systems is crucial to evaluate performance under such heterogeneity. Additionally, secondary pollution risks, such as residual Fe leaching or biochar-bound toxic intermediates, require mitigation strategies, such as post-treatment filtration or material regeneration. Recent studies suggest that magnetic biochar-ZVI composites enable easy recovery, addressing both cost and environmental safety concerns.

## 4. Conclusions

This study systematically elucidates the catalytic role of organic amendments (HA/OA) in enhancing THI removal by ZVI and ZVI/PBC systems, validated through multi-model characterization (XRD, XPS, and electrochemical analysis) and mechanistic modeling. HA and OA significantly reduce iron oxide passivation on ZVI surfaces, with OA exhibiting superior performance due to its low molecular weight, enabling deeper interfacial penetration and stronger Fe^2+^ chelation. This enhancement was amplified by the ZVI/PBC composite, which achieved a THI removal efficiency of 73.44% in the OA-modified system and a ~2-fold improvement over the pristine ZVI. Organic acid coordination replaces passivating Fe–OH/Fe–O bonds with labile Fe–COO complexes (28.61% surface coverage), reducing charge transfer resistance by 58% and sustaining Fe^0^/Fe^2+^ redox cycling. These work insights establish a structure–activity framework linking organic ligand properties (molecular size and chelation capacity) to catalytic efficiency, providing critical design principles for optimizing iron-based materials in pesticide remediation. Furthermore, this work pioneers a mechanistic understanding of soil organic matter interactions with engineered composites, informing field-scale strategies for mitigating agrochemical contamination in heterogeneous environments.

To advance this research toward practical implementation, future efforts should focus on the following actionable directions:

1. Optimizing material synthesis protocols, such as low-temperature pyrolysis for biochar or green reductants (e.g., plant extracts) for ZVI production, to enhance cost-effectiveness and scalability.
2. Expanding application scenarios by evaluating system performance under diverse environmental conditions (e.g., saline soils and organic-rich wastewater) and co-contaminant matrices (e.g., heavy metals and microplastics).
3. Developing real-time monitoring platforms integrating in situ spectroscopy (e.g., Raman) or artificial intelligence (AI)-driven sensor networks to track degradation intermediates and catalyst stability dynamically.
4. Engineering recoverable composites, such as magnetic ZVI/PBC hybrids, to minimize secondary pollution and facilitate large-scale deployment.

## Figures and Tables

**Table 1:** Degradation efficiency of THI by different catalytic degradation materials.

Catalytic Degradation Materials	Additional Conditions	Degradation Efficiency	Reference
PTsPS composite aerogel	Solar light	42%	[33]
Chlorine	/	9.17%	[34]
UV	/	52.96%	
Chlorine	UV	77.68%	
Sludge biochar (SBC)	/	19.17%	[35]
SBC	Periodate	28.06%	
Fe-SBC	/	52.55%	
	Periodate	69.72%	
Mn-SBC	/	39.20%	
	Periodate	41.91%	
Fe/Mn-SBC	/	26.59%	
	Periodate	84.92%	
Fe-doped MXene (MXF)	UV	21.51%	[36]
MXF with added ascorbic acid (MXF_AA_)	UV	27.01%	
Fe-based metal–organic frameworks (MIL(Fe))	UV	40.38%	
MXF/MIL(Fe)	UV	51.76%	
MXF_AA_/MIL(Fe)	UV	64.63%	
PBC	/	9.09%	This work
ZVI	/	19.23%	
ZVI/PBC	/	36.65%	
ZVI/PBC	HA	39.90%	
ZVI/PBC	OA	74.16%	

## Data Availability

The original contributions presented in this study are included in the article. Further inquiries can be directed to the corresponding author.
